# Determinants of Recycling Behavior in Emerging Waste Management Systems: The Role of Environmental Concern, Knowledge, Moral Norms, and Attitude

**DOI:** 10.3390/bs16071185

**Published:** 2026-07-14

**Authors:** Almukhtar Aljatlawe, Askin Kiraz

**Affiliations:** Environmental Education and Management, Near East University, Nicosia 99138, North Cyprus, Turkey; askin.kiraz@neu.edu.tr

**Keywords:** recycling behavior, waste management systems, circular economy, environmental concern, recycling knowledge, moral norms, attitudes toward recycling

## Abstract

The effectiveness of waste recycling systems in developing contexts is constrained not only by limited infrastructure but also by insufficient behavioral engagement. This study advances the understanding of recycling behavior by proposing and testing an integrated framework that investigated the combined effects of environmental concern, recycling knowledge, and moral norms, as well as their indirect effects through attitudes toward recycling. Using a cross-sectional survey administered on 516 university students in Libya, the study employed regression and mediation analyses. The results demonstrate that recycling knowledge is the most significant predictor of attitudes toward recycling, while attitudes serve as a critical mechanism driving recycling behavior. Environmental concern and moral norms were found to exert significant direct and partially mediated effects, revealing multiple pathways through which these factors influence recycling behavior. These findings provide empirical support for a multidimensional explanation of recycling behavior in which cognitive, normative, and attitudinal factors jointly shape outcomes. This study provides evidence from an underexplored context and extends existing models of pro-environmental behavior and highlights the role of knowledge-driven and attitude-based interventions. The findings provide actionable insights for designing behavior-focused interventions to enhance recycling participation and improve sustainable waste management outcomes in developing regions.

## 1. Introduction

Municipal waste generation continues to grow globally, placing severe strain on municipal waste management systems and public health infrastructures (Nurzhan et al., 2025). Recycling is widely recognized as an efficient approach for sustainable waste management, because it has the potential to significantly reduce waste generation, conserve natural resources, and support the transition toward a circular economy (Kaza et al., 2018). However, recycling performance is not only determined by waste management infrastructure, but it also critically depends on individuals’ consistent engagement in recycling-oriented waste management behaviors (Steg & Vlek, 2009; Kollmuss & Agyeman, 2002). Hence, recycling is recognized as a pro-environmental behavior which is shaped by both social and psychological determinants, rather than only technical waste management practices (Ajzen, 1991; Tonglet et al., 2004).

### 1.1. Research Background

Waste management and recycling pose a complex challenge in developing and transitional contexts, due to factors such as limited infrastructural systems, inconsistent environmental regulations, and inadequate environmental concern (Fayid & Kiraz, 2023). Libya provides a relevant case for examining recycling behavior due to the socioeconomic constraints and institutional disruptions of the country; these have significantly limited waste management infrastructure including recycling (Alzayani et al., 2025). Under such conditions, the participation of the public in recycling is poorly institutionalized and primarily relies on strong individual motivation, moral obligation, and knowledge-based capability (Alsadey & Hamid, 2022).

University students represent a strategically relevant group for global sustainability research and intervention, because they are in a developmental stage of long-term habit formation (Biancardi et al., 2023). Students play important roles as future decision-makers and in shaping pro-environmental norms in their respective communities. Institutions of higher education represent influential sites for sustainability education, environmental campaigns, and policy-driven behavioral cues (Vicente-Molina et al., 2013; Zsóka et al., 2013; Lozano et al., 2015). Accordingly, investigating the recycling behaviors among Libyan university students can provide valuable evidence to guide effective educational and institutional strategies toward recycling and environmental sustainability.

### 1.2. Research Aim and Objectives

Previous studies have independently examined the relationships between environmental concern, recycling knowledge, moral norms, and pro-environmental behavior. However, relatively few studies have examined these determinants simultaneously within an integrated framework that considers both their direct effects on recycling behavior and their indirect effects through attitudes towards recycling. Existing research has also been conducted predominantly in developed or rapidly industrializing countries, leaving developing and post-conflict contexts such as Libya largely underrepresented in the literature. Consequently, it remains unclear whether the mechanisms underlying recycling behavior observed in other contexts are equally applicable in environments characterized by limited waste management infrastructure and weak institutional support.

University students represent an important population for investigating recycling behavior because they are future professionals, policymakers, and community leaders who are likely to influence environmental practices over the long term. Universities also provide environments in which environmental values, sustainability knowledge, and civic responsibility are actively developed, making students an appropriate population for examining the psychological determinants of recycling behavior.

## 2. Theoretical Foundations

### 2.1. Recycling as a Pro-Environmental Action

Recycling is primarily framed as a key aspect of pro-environmental behavior, because it supports resource conservation and reduces environmental deterioration caused by waste generation (He et al., 2024; Saulītis et al., 2024). Participation in recycling behavior is, however, dependent on multiple factors, including positive perceptions of recycling, understanding the required actions, and having sufficient motivation to engage in it (Ma et al., 2023; Mohamad et al., 2022). The literature emphasizes the psychological and cognitive predictors of recycling, rather than considering only infrastructural dependencies of recycling (Adjei et al., 2023; Liu et al., 2022; Bao et al., 2023). This study conceptualizes recycling behavior as student engagement in recycling-related actions toward environmental sustainability.

In addition to its benefits, recycling behavior contributes to the operational effectiveness of waste recycling systems by improving source separation and reducing contamination in recyclable materials. These behavioral contributions are essential for optimizing the efficiency of recycling technologies and supporting sustainable material cycles.

### 2.2. Attitude Toward Recycling as a Key Behavioral Determinant

Attitude toward a behavior is commonly defined as an individual’s overall evaluation of engaging in that behavior (Rozenkowska, 2023). According to the Theory of Planned Behavior (TPB), attitude plays a critical role in shaping individual intention and actual behavioral engagement (Ajzen, 1991). In the context of recycling, positive attitudes toward recycling have been strongly associated with engaging in recycling-related pro-environmental behaviors (Miller et al., 2022; Kerse & Kerse, 2025; Nguyen et al., 2025; Zuo et al., 2025). Attitudes are particularly important for behaviors that require sustained effort and repeated engagement, individuals who perceive recycling positively and with considerable benefits are more likely to engage in sustained recycling behavior (Lim et al., 2025).

**H1.**  *Attitudes toward recycling have a significant effect on recycling behavior.*

### 2.3. Environmental Concern and Attitude Toward Recycling

Environmental concern is characterized as the individual’s concern toward environmental conditions, and their beliefs about human and environment interactions (Si et al., 2022). Ecological worldview perspectives emphasize that individuals who recognize environmental risks and have adequate perceptions of the vulnerability of the environment are more likely to support pro-environmental behaviors (Kasperson et al., 2022). Environmental concern may promote recycling behavior by increasing the perceived importance of recycling and strengthening positive evaluations as an environmentally responsible practice (Chao et al., 2023). Environmental concern has consistently been identified as one of the strongest antecedents of pro-environmental behavior. Individuals with stronger concern for environmental degradation are generally more willing to support environmentally responsible actions, including recycling, energy conservation, and sustainable consumption. Prior studies have shown that environmental concern increases perceived responsibility toward environmental protection and encourages individuals to adopt behaviors that minimize ecological harm (Wibowo et al., 2023).

The New Ecological Paradigm (NEP) framework conceptualizes environmental concern as a broader ecological worldview reflecting beliefs about the relationship between humans and nature (Dunlap et al., 2000). Individuals who endorse stronger ecological worldviews tend to express greater support for environmental protection and sustainable waste management practices (Mishra et al., 2023). In the context of recycling behavior, environmental concern may motivate individuals to view recycling as a meaningful contribution toward environmental preservation and resource conservation (Mannoni, 2025).

**H2.**  *Environmental concern has a positive effect on attitudes toward recycling behavior.*

### 2.4. Recycling Knowledge and Attitude Toward Recycling

Recycling knowledge refers to the knowledge required for individuals to understand recycling as a process, including the identification of recyclable and reusable materials (Holt et al., 2023). Awareness and positive perceptions of recycling without adequate knowledge on the process significantly challenges and restricts participation by individuals (Owojori et al., 2022). A lack of recycling knowledge restricts individual participation in recycling, increases perceived efforts due to uncertainties, and reduces confidence and self-efficacy (Noh, 2024). Knowledge influences individual attitudes because it enables individual recycling evaluation positively (Yadav et al., 2022), adequate recycling knowledge also increases perceived self-efficacy and positive outcomes (Baldwin et al., 2023).

The relationship between recycling knowledge and recycling behavior has received increasing attention in environmental behavior research. Knowledge reduces uncertainty regarding recycling procedures and enhances individuals’ confidence in their ability to participate effectively (Mendoza et al., 2025). Studies have demonstrated that individuals who possess greater knowledge regarding recyclable materials, waste-sorting procedures, and recycling benefits are more likely to develop favorable evaluations of recycling activities (Mago et al., 2025).

Furthermore, recycling knowledge facilitates the translation of environmental concern into concrete action. Even when individuals express concern for environmental issues, insufficient knowledge regarding recycling processes may prevent participation (Mi et al., 2026). Consequently, knowledge functions not only as a cognitive resource but also as a practical mechanism supporting behavioral engagement.

**H3.**  *Recycling knowledge has a positive effect on attitudes toward recycling.*

### 2.5. Moral Norms and Attitude Toward Recycling

Moral norms are an individual’s internalized sense of moral obligations with regard to whether to engage in a particular action (Krettenauer, 2022). Pro-environmental behaviors such as recycling behaviors are often associated with perceived responsibility toward society and future generations (Skeirytė et al., 2022). Moral norms are considered critical psychological determinants for behavioral actions such as pro-environmental behaviors (van Valkengoed et al., 2022). According to normative models of pro-social behavior, individual perception of responsibility toward an action and perceptions of guilt in failing to do so increases the likelihood of behavioral engagement (Scaffidi Abbate et al., 2022). Moral norms increase the likelihood of pro-environmental behavioral engagement; this connects it to self-expectations and ethical consistency (Silva et al., 2025).

Moral norms differ from general environmental attitudes because they reflect a personal sense of obligation rather than favorable evaluation. Individuals may participate in recycling not only because they perceive benefits from activity but also because they believe recycling represents the moral obligation to recycle, which frequently predicts environmentally responsible behaviors even when external incentives are absent (Islam et al., 2025).

The influence of moral norms may be particularly important in contexts where recycling infrastructure remains underdeveloped. In such situations, participation often depends less on institutional support and more on individuals’ internal sense of responsibility toward environmental sustainability and future generations (Furness et al., 2025).

**H4.**  *Moral norms have a positive effect on attitudes toward recycling.*

### 2.6. The Mediating Role of Attitude Toward Recycling in Recycling Behavior

The conceptual framework proposed in this study assumes that environmental concern, recycling knowledge, and moral norms are distinct antecedent variables that influence recycling behavior directly and indirectly through attitudes toward recycling. These constructs are conceptualized as independent psychological determinants rather than components of a general pro-environmental attitude. Within this framework, attitude toward recycling functions as the mediating mechanism linking these antecedent variables to recycling behavior.

Awareness of the environmental consequences of environmental deterioration, such as inadequate waste management (Abubakar et al., 2022), adequate recycling knowledge (Ding et al., 2023), and perceptions of ethical responsibility toward environmental responsibility defined by moral norms (Tsai & Tan, 2022) often contribute to the development of recycling behavior. This theoretical approach is consistent with the TPB framework reasoning, where attitudes serve as a critical psychological pathway between underlying beliefs and behavioral outcomes (Hamilton & Hagger, 2025; Hagger, 2025).

**H5.**  *Attitude toward recycling mediates the relationship between environmental concern and recycling behavior.*

**H6.**  *Attitude toward recycling mediates the relationship between recycling knowledge and recycling behavior.*

**H7.**  *Attitude toward recycling mediates the relationship between moral norms and recycling behavior.*

**Figure 1:**
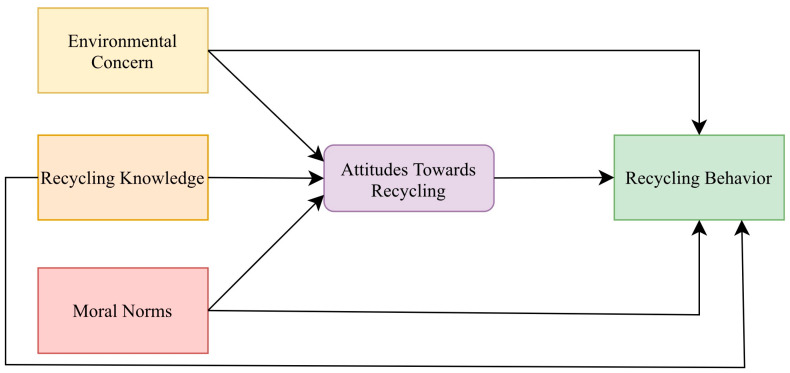
Research framework.

This study adopted a framework which allows the research to investigate both direct and mediating roles of attitudes in predicting behavior. The study investigated the extent to which attitude serves as a mediating mechanism linking foundational predictors of behavioral outcome in the context of recycling behaviors in university students. Figure 1 illustrates the research framework proposed and investigated in this study.

## 3. Methodology

### 3.1. Research Design

This study adopted a quantitative cross-sectional survey design to carry out an investigation into the relationships between environmental concern, recycling knowledge, moral norms, attitude toward recycling, and recycling behavior among university students in Libya. The study used a survey approach, deeming it appropriate to measure latent psychological constructs on self-reported behavior, and to test theoretically grounded relationships among the variables in the context of this study.

The study used validated multi-item scales to measure the constructs of the study, which is followed by both descriptive and bivariate analyses to explore the socio-demographic characteristics of the study participants. Finally, regression analysis was used to test direct and indirect relationships between the research variables to test the formulated research hypotheses.

This study also considers the broader implications of behavioral determinants for waste management systems, particularly in contexts where technological infrastructure is limited and behavioral engagement plays a central role in recycling effectiveness.

### 3.2. Study Case and Sample

In recent decades, Libya has increasingly experienced severe environmental deterioration. This is reflected in the growing waste accumulation, land degradation, and increased pressure on the already fragile ecological system (Ziadat et al., 2025). Rapid urbanization, changing consumption patterns and limited environmental regulation, exacerbated by the prolonged civil conflict, continue to contribute substantially to the visible environmental stress in the country (Alsharif et al., 2025). A significant contributor to environmental deterioration in Libya is the inadequacy of waste management systems, characterized by insufficient waste segregation, recycling, and poorly managed disposal systems (Fayid & Kiraz, 2023). These structural weaknesses intensify environmental deterioration and public health risks, they also reduce opportunities for citizens’ engagement in environmentally responsible practices such as recycling.

Participants were recruited using a convenience sampling strategy from Libyan universities. Survey invitations were distributed through institutional communication channels and student academic networks. Eligibility criteria required respondents to be currently enrolled university students and at least 18 years of age. Participation was voluntary and no incentives were provided. A total of 516 valid responses were retained for analysis after screening for completeness.

This study is focused on Libya’s university students, a group positioned at the intersection of education, social influence, and emerging civic responsibility. Universities represent micro-environments where environmental concern, knowledge, and moral responsibility are often cultivated despite systemic limitations. Investigating this group provides insight into how psychological factors shape recycling behavior in a context where institutional recycling structures remain underdeveloped. The student population also offers a meaningful case to explore the determinants of recycling as an environmentally responsible behavior within Libya’s broader environmental and waste management challenges.

### 3.3. Data Collection Procedure

Data were collected using a structured self-administered questionnaire distributed electronically through institutional and academic communication channels. Before participation, respondents were provided with information describing the study objectives, voluntary nature of participation, and ethical considerations. Participants completed the questionnaire independently and were informed that no personally identifying information will be collected. Only fully completed questionnaires were included in the final analysis.

### 3.4. Data Collection Tool

The research questionnaire used in this study was structured into several sections, with each section corresponding to a key construct in the study’s research framework. All constructs were measured using previously validated instruments. Environmental concern and moral norms were measured using seven-point Likert scales ranging from 1 (strongly disagree) to 7 (strongly agree). Recycling knowledge and attitudes toward recycling were measured using five-point Likert scales ranging from 1 (strongly disagree) to 5 (strongly agree). Recycling behavior was measured using a seven-point frequency scale ranging from 1 (never) to 7 (always). Composite construct scores reported in the analyses represent the mean responses across all items comprising each construct.

Environmental concern was measured using items derived from the New Ecological Paradigm (NEP) scale originally developed by Dunlap et al. (2000), and subsequently adapted in studies examining environmental attitudes and ecological worldviews, including Ntanos et al. (2019). The scale contains 15 items measuring ecological concern and beliefs regarding human-environmental relationships. Recycling attitude and recycling knowledge were measured using scales adapted from Asri and Daud (2022); the recycling attitude scale contains five items, where each item is used to measure the overall orientation of respondents toward recycling as they perceive it. The scale used to measure recycling knowledge contains a total of five items, where each item measures respondents’ knowledge on carrying out recycling practices. Recycling behaviors were measured using a scale adapted from Osman et al. (2014); this scale contains a total of eight items, with each item evaluating respondents’ extent of engaging in recycling behaviors. Moral norms were measured using a scale adapted from Wan et al. (2014); this scale contains a total of five items, where each item is used to capture respondents’ internalized sense of responsibility and ethical obligation toward recycling.

Table 1 presents the demographic characteristics of the research respondents. Male students represented 50.7% of the sample, while female students represented 49.3% of the sample. The majority of the students were between 21 and 25 years of age. These characteristics indicate that the sample broadly reflects the typical demographic composition of undergraduate university students in Libya.

### 3.5. Data Reliability and Validation

After data collection, data completeness and consistency were tested to ensure statistical rigor. All items requiring reverse coding were transformed accordingly, and composite scores were calculated for each construct by averaging corresponding items. Internal consistency for each scale was assessed using Cronbach’s alpha, with values of 0.70 or higher considered acceptable. Statistical significance was evaluated at the 5% level (*p* < 0.05). Table 2 shows the internal consistency of the research scales.

The internal consistency of all the study’s measurement scales as reported in Table 2 indicate that all research constructs exhibited acceptable and reliable measurements. All the Cronbach’s alpha coefficients exceeded 0.7 as the minimum acceptable value for internal consistency.

### 3.6. Assessment of Common Method Variance

Because data for all study variables were collected using a single self-administered questionnaire, the potential influence of common method variance (CMV) was assessed using Harman’s single-factor test (Podsakoff et al., 2003). An unrotated principal component analysis including all measurement items was conducted. The first unrotated factor accounted for 21.01% of the total variance, which is substantially below the commonly accepted threshold of 50%. Therefore, the findings suggest that CMV is unlikely to have significantly influenced the observed relationships among the study variables.

## 4. Results

Table 3 presents the descriptive statistics for the study constructs. Because composite mean scores were calculated, the observed maximum values represent respondents’ average item scores rather than the maximum possible score for individual questionnaire items. All construct means fall within the theoretical ranges of their respective measurement scales, indicating no anomalies in the observed data distribution.

Table 4 shows the results of an independent sample *t*-test examining the gender differences in the study participants across the research constructs. The results indicated no statistically significant difference in gender responses for environmental concern, indicating a similar level of environmental concern among both male and female respondents.

Recycling knowledge, moral norms, attitude toward recycling, and recycling behavior all showed significant differences between the male and female respondents. Female respondents exhibited more positive attitudes toward recycling, and significantly higher levels of recycling behavior compared to the male respondents. These findings suggest that while general environmental concern is comparable among the respondents, female respondents show stronger normative, cognitive, and behavioral engagement in recycling.

Table 5 presents results for independent-samples t-tests between age groups on each research construct. Because only eleven respondents were aged 26 years or older, these age categories were excluded from the age comparison to avoid unreliable statistical inference arising from extremely small subgroup sizes. Independent-sample *t*-tests were therefore conducted to compare respondents aged 18–20 years with those aged 21–25 years. The results showed no statistically significant differences between the two age groups for environmental concern, recycling knowledge, moral norms, attitude towards recycling or recycling behavior. These findings indicate that recycling-related attitudes, knowledge, moral norms, environmental concern, and recycling behavior were generally consistent across the two principal age groups represented in the study.

Table 6 presents the results of the Pearson correlation analysis; the correlation coefficients show statistically significant and positive correlations among all the research constructs at the 0.01 significance level. Strong correlations were found between recycling knowledge and moral norms, and a moderate correlation was found between recycling knowledge, moral norm, and recycling behavior. Moral norms also exhibited a strong and positive correlation with recycling behavior.

Attitude toward recycling showed a moderate and positive correlation with recycling behavior, indicating that favorable recycling attitudes are associated with high engagement in recycling behavior. Environmental concern showed positive correlation with all constructs; the overall correlation findings provide preliminary support for further tests for hypothesized relationships for direct and indirect impacts between the research constructs.

Table 7 shows the results of a multiple regression analysis carried out to examine the impacts of environmental concern, recycling knowledge, and moral norms on attitude toward recycling. The results of this regression analysis show recycling knowledge as the strongest predictor of attitude toward recycling with β = 0.330 and *p* < 0.001. Both moral norms and environmental concern also show positive but statistically non-significant effects on attitudes toward recycling. These findings suggest recycling knowledge plays a significant role in shaping attitudes toward recycling among individuals, and while awareness and moral norms have positive influences, they have a limited direct impact on attitudes toward recycling.

**H2.**  *Environmental concern has a positive effect on attitudes toward recycling behavior—not supported.*

**H3.**  *Recycling knowledge has a positive effect on attitudes toward recycling—supported.*

**H4.**  *Moral norms have a positive effect on attitudes toward recycling—not supported.*

Table 8 shows results of a simple regression analysis carried out to test the effect of attitude toward recycling on recycling behavior. The results show attitudes toward recycling have a significant and positive effect on recycling behavior with β = 0.411 and *p* < 0.001. The results indicate that individuals with more positive attitudes toward recycling are more likely to engage in recycling behaviors.

**H1.**  *Attitudes toward recycling have a significant effect on recycling behavior—supported.*

**Figure 2:**
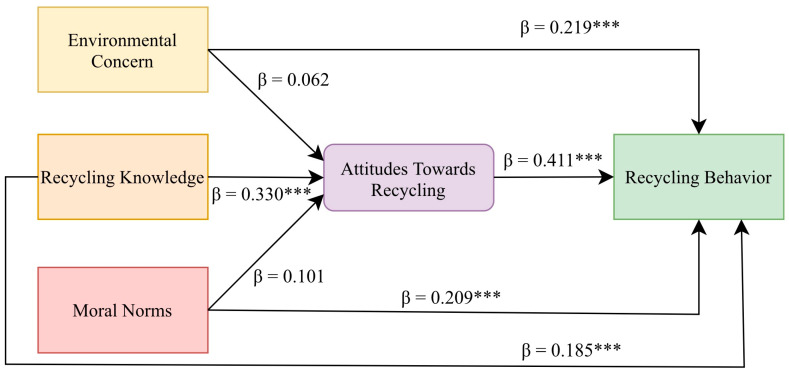
Summary of standardized regression coefficients illustrating the direct and indirect relationships among the study variables. Note. Values represent standardized regression coefficients (β). *** = *p* < 0.001.

The regression analysis as shown in Table 9 demonstrates that environmental concern, recycling knowledge, moral norms, and attitudes toward recycling each exert significant positive effects on recycling behavior. Among these predictors, attitudes toward recycling exhibited the strongest standardized effect, highlighting the role in promoting recycling behavior. Figure 2 provides a summary of the direct relationships among the study variables together with the mediating role of attitudes toward recycling. The coefficients presented in the figure were obtained from separate regression and mediation analyses and are intended as a conceptual summary rather than as a structural equation model.

Although environmental concern and moral norms did not exhibit statistically significant direct effects on attitudes towards recycling in the multiple regression model, the bootstrapped indirect effects remained statistically significant because their confidence intervals excluded zero. Contemporary mediation analysis recommends evaluating mediation using the significance of the bootstrapped indirect effect rather than relying solely on using the significance of the individual regression paths (Zhao et al., 2010; Hayes, 2017).

The mediating role of attitudes toward recycling was examined using bootstrapping procedures, as shown in Table 10. The indirect effects of environmental concern, recycling knowledge, and moral norms via attitudes toward recycling were all found to be statistically significant, as none of the confidence intervals included zero. These results indicate that attitude toward recycling serves as a significant mediating mechanism linking environmental concern, recycling knowledge, and moral norms to recycling behavior.

The direct effects of these predictors on recycling behavior remained statistically significant as shown in Table 8, suggesting mediation is partial rather than full. The overall findings in this study demonstrate that recycling behavior is influenced directly and indirectly via attitudes toward recycling, highlighting the combined role of cognitive, normative, and attitudinal factors in shaping pro-environmental behavior.

**H5.**  *Attitude toward recycling mediates the relationship between environmental concern and recycling behavior—supported.*

**H6.**  *Attitude toward recycling mediates the relationship between recycling knowledge and recycling behavior—supported.*

**H7.**  *Attitude toward recycling mediates the relationship between moral norms and recycling behavior—supported.*

## 5. Discussion

This study investigated the determinants of recycling behavior by examining the roles of environmental concern, recycling knowledge, moral norms, and attitudes toward recycling. The present findings have provided insight into the subject of recycling behavior within the Libyan context.

### 5.1. Determinants of Attitudes Toward Recycling

Among the variables examined as predictors of attitudes toward recycling, recycling knowledge emerged as the strongest and most statistically significant predictor. This finding underscores the critical role of pro-environmental knowledge in shaping individual evaluations toward pro-environmental behavior such as recycling behavior. This finding suggests that individuals with an adequate understanding of recycling processes, their benefits, and practical procedures required for recycling are more likely to develop positive attitudes toward recycling. This finding aligns with previous research emphasizing the importance of domain specific knowledge in shaping environmental attitudes and behavioral intentions (Kaiser & Fuhrer, 2003; Frick et al., 2004; Vicente-Molina et al., 2013).

Environmental concern was not found to have a statistically significant direct effect on attitude toward recycling, even though there was an observed positive relationship. This suggests that general awareness of environmental trends may be insufficient to influence attitudes toward specific pro-environmental behaviors, such as attitudes toward recycling. Previous studies have reported that abstract environmental concern does not always translate to favorable environmental attitudes, unless it is accompanied by actionable knowledge (Kollmuss & Agyeman, 2002; Bamberg & Möser, 2007). In the Libyan context, where recycling systems and infrastructure remain underdeveloped, environmental concern may not readily translate into favorable attitudes toward recycling.

The absence of significant direct effects of environmental concern and moral norms on attitudes toward recycling, despite their significant direct effects on recycling behavior, suggests that these variables may influence behavior through mechanisms other than attitudes. This finding is consistent with the attitude–behavior gap described by Colombo et al. (2023), whereby individuals may engage in environmentally responsible behaviors despite expressing only moderate attitudes toward specific environmental actions. It is also compatible with the Value Belief Norm theory (Stern et al., 1999), which proposes that personal values and moral obligations can motivate pro-environmental behavior independently of attitudinal evaluations. Within the Libyan context, where recycling opportunities remain limited and institutional support is relatively weak, individuals may rely more heavily on personal environmental values and moral responsibility than on favorable attitudes toward recycling itself.

The present findings indicate that moral norms did not have a statistically significant effect on attitudes toward recycling, although the positive coefficient suggests a weak directional relationship. This finding is consistent with norm activation theory, which proposes that moral norms often exert indirect influences on attitudes, particularly in contexts where behavioral norms are not strongly institutionalized (Liao, 2025; Marchetti et al., 2025).

### 5.2. Determinants of Recycling Behavior

The relationship between attitude toward recycling and recycling behavior has been found to be consistent with theoretical expectations. The finding supports the TPB proposition which posits favorable attitudes toward a behavior, and increases the likelihood of engaging in the behavior (Ajzen, 1991). In the Libyan context, the result suggests individuals with a perception of recycling being beneficial are more likely to engage in the practice, even in the absence of fully developed waste management infrastructure. Hence, this reinforces the importance of attitudinal change as a critical factor in promoting pro-environmental behaviors in developing regions.

The study found a significant impact of environmental concern, recycling knowledge, and moral norms on recycling behavior, indicating recycling behavior is influenced via other pathways, such as cognitive factors, moral obligation, and environmental concern. This is consistent with studies reporting pro-environmental behaviors being driven by a combination of critical factors: instrumental, normative, and affective factors (Stern, 2000; Thøgersen, 2006; Steg & Vlek, 2009). Attitude toward recycling was found to have a partial mediation between the analyzed independent factors on recycling behavior; this aligns with integrated behavioral models that acknowledge multiple routes to action: attitude, habitual, and normative (Bamberg, 2003; Klöckner et al., 2025). In the context of Libya where recycling infrastructure and policy enforcement are still emerging, individuals are more likely to rely on personal and moral reasoning to guide behavior toward pro-environmental behavior such as recycling.

The present findings also illustrate that psychological determinants of recycling behavior may operate differently in resource-constrained settings than in highly institutionalized recycling systems. In Libya, recycling opportunities are often constrained by limited collection infrastructure, weak regulatory enforcement, and inconsistent environmental policies. Under these circumstances, behavioral decisions may depend less on favorable attitudes toward recycling and more on an individual’s environmental values, perceived moral obligations, and practical knowledge. Consequently, educational interventions aimed at strengthening recycling competence may be more effective than campaigns that focus exclusively on changing attitudes.

From a systems perspective, these findings highlight that effective waste recycling is not solely dependent on technological infrastructure but also on behavioral readiness. In developing waste management systems, individual recycling behavior directly influences the quality and quantity of recyclable materials, thereby affecting the efficiency of downstream recycling technologies. Enhancing recycling knowledge and attitudes can therefore improve the overall performance of recycling systems. Importantly, the findings demonstrate that behavioral determinants operate alongside structural constraints, reinforcing the need for integrated behavioral and system level interventions.

### 5.3. Theoretical and Contextual Implications

The findings in this study contribute to the research literature by demonstrating that recycling knowledge plays a critical role in shaping attitudes toward recycling, while attitude serves as a key pathway to recycling behavior. From a contextual perspective, the findings highlight the importance of knowledge-based and norm-based interventions in promoting recycling behavior in Libya. Given the limited development of general waste management and recycling infrastructure, behavioral change may be more effectively driven using educational initiatives and awareness campaigns that emphasize practical recycling knowledge and promote the moral appeal of recycling. This study demonstrates that recycling behavior is shaped by an interplay between several factors: cognitive normative, and attitudinal. Recycling knowledge emerges as the most impactful driver of attitudes toward recycling; attitude significantly predicts recycling behavior, and also mediates the effects of awareness, knowledge and moral norms.

The Libyan context also contributes theoretically by demonstrating that pro-environmental behavior models developed primarily in industrialized countries remain broadly applicable while also highlighting important contextual differences. Specifically, the findings suggest that environmental concern and moral norms may influence recycling behavior through pathways that are less dependent on attitudinal evaluations when institutional recycling systems are underdeveloped. These results support calls for greater contextualization of behavioral theories within developing and post-conflict environments.

### 5.4. Implications for Waste Recycling Systems and Circular Economy

The findings have important implications for waste recycling systems and circular economy development. Behavioral determinants, particularly recycling knowledge and moral norms, play a critical role in promoting effective source separation, which is fundamental to efficient recycling systems. In contexts with limited technological infrastructure, strengthening behavioral engagement can enhance material recovery rates and reduce contamination in recyclable streams. Furthermore, integrating behavioral interventions with technological advancements can support the transition toward circular economy models, where waste materials are re-introduced into production systems.

## 6. Conclusions

This study examined the determinants of recycling behavior by analyzing environmental concern, recycling knowledge, moral norms, and attitudes toward recycling using an integrated framework. The findings of the study indicate that recycling behavior is shaped by multiple complementary factors and mechanisms, with recycling knowledge as the strongest predictor of attitudes toward recycling and attitude serving as a critical driver of behavior. Environmental concern and moral norms were found to have both direct and indirect effects on recycling behavior, with attitudes toward recycling as a partial mediator, indicating that these factors influence behavior via attitudinal and non-attitudinal factors. The findings highlight the importance of knowledge-based pro-environmental interventions, attitude-focused interventions, and campaigns that promote a stronger sense of moral responsibility for pro-environmental behaviors, such as recycling. These factors play a critical role in enhancing pro-environmental behavior particularly in developing countries with limited waste management infrastructure, such as Libya. This study further emphasizes that strengthening behavioral drivers of recycling is essential for improving the effectiveness of waste recycling systems and supporting circular economy initiatives, particularly in developing regions with limited waste management infrastructure.

### Recommendation and Future Work

Based on the findings of this study, the following practical recommendations are proposed: policymakers and educational institutions should prioritize domain-specific pro-environmental behavior education and knowledge dissemination. Attitude-focused campaigns should be communicated to emphasize narratives of individual and collective duty toward environmental sustainability. These recommendations would help ensure that the development of recycling infrastructure is complemented by individual and collective efforts toward environmental sustainability. Future work should adopt longitudinal research design and incorporation of contextual factors such as infrastructural constraints and perceived behavioral control. Such research would further advance understanding of recycling behavior and pro-environmental behavior more broadly.

## Figures and Tables

**Table 1:** Research sample demographic characteristics.

Variable	Category	Frequency	Percentage (%)
Gender	Male	262	50.7
	Female	255	49.3
Age range	18 to 20 years	112	21.7
	21 to 25 years	394	76.2
	26 to 30 years	8	1.5
	31 years and above	3	0.6

**Table 2:** Reliability analysis.

Construct	Cronbach’s Alpha (α)
Environmental concern	0.781
Recycling knowledge	0.709
Moral norms	0.794
Attitude toward recycling	0.762
Recycling behavior	0.818

**Table 3:** Constructs’ descriptive statistics.

	N	Minimum	Maximum	Mean	Std. Deviation
Environmental concern	516	2.60	6.13	4.4434	0.66935
Recycling knowledge	516	1.00	5.00	3.7791	0.68177
Moral norms	516	1.60	7.00	4.6694	1.29474
Attitude toward recycling	516	2.20	5.00	3.6236	0.58338
Recycling behavior	516	1.88	7.00	5.2347	1.06928
Valid N (listwise)	516				

**Table 4:** Gender differences in recycling-related constructs.

Construct	Gender	Mean	Std. Deviation	*t*	*p*
Environmental Concern	Male	4.41	0.67	1.28	0.201
	Female	4.47	0.66		
Recycling Knowledge	Male	3.71	0.69	2.94	0.003
	Female	3.85	0.66		
Moral Norms	Male	4.52	1.33	2.11	0.035
	Female	4.78	1.25		
Attitude Toward Recycling	Male	3.55	0.60	2.47	0.014
	Female	3.70	0.56		
Recycling Behavior	Male	5.07	1.11	3.62	<0.001
	Female	5.39	1.01		

**Table 5:** Age group differences in recycling-related constructs between respondents aged 18–20 years and 21–25 years.

Construct	Age Group	Mean	Std. Deviation	*t*	*p*
Environmental Concern	18–20 years	4.39	0.70	−1.03	0.302
	21–25 years	4.46	0.67		
Recycling Knowledge	18–20 years	3.77	0.72	−0.16	0.870
	21–25 years	3.78	0.68		
Moral Norms	18–20 years	4.57	1.47	−0.83	0.406
	21–25 years	4.70	1.25		
Attitude Toward Recycling	18–20 years	3.67	0.66	1.04	0.299
	21–25 years	3.61	0.57		
Recycling Behavior	18–20 years	5.38	1.24	1.34	0.181
	21–25 years	5.21	1.01		

**Table 6:** Correlation matrix table.

Variable	1	2	3	4	5
1. Environmental concern	-				
2. Recycling knowledge	0.299 **	-			
3. Moral norms	0.285 **	0.570 **	-		
4. Attitude toward recycling	0.189 **	0.406 **	0.307 **	-	
5. Recycling behavior	0.377 **	0.463 **	0.447 **	0.411 **	-

Pearson correlation coefficients are reported. ** indicates *p* < 0.01 (two-tailed).

**Table 7:** Regression analysis for factors predicting attitude toward recycling.

Predictor	B	SE	β	*t*	*p*
Environmental Concern	0.054	0.031	0.062	1.74	0.082
Recycling Knowledge	0.282	0.031	0.330	9.10	<0.001
Moral Norms	0.046	0.024	0.101	1.92	0.055

B = unstandardized regression coefficient; SE = standard error; β = standardized regression coefficient.

**Table 8:** Simple regression analysis for attitude toward recycling predicting recycling behavior.

Predictor	B	SE	β	*t*	*p*
Attitude Toward Recycling	0.754	0.066	0.411	11.47	<0.001

B = unstandardized regression coefficient; SE = standard error; β = standardized regression coefficient.

**Table 9:** Regression analysis for factors predicting recycling behavior.

Predictor	B	SE	β	*t*	*p*
Environmental Concern	0.350	0.053	0.219	6.60	<0.001
Recycling Knowledge	0.290	0.057	0.185	5.09	<0.001
Moral Norms	0.173	0.029	0.209	5.98	<0.001
Attitude Toward Recycling	0.421	0.063	0.230	6.68	<0.001

B = unstandardized regression coefficient; SE = standard error; β = standardized regression coefficient.

**Table 10:** Mediation analysis for indirect effects of attitude toward recycling on recycling behavior.

Predictor (X)	Mediator (M)	Outcome (Y)	Indirect Effect (β)	BootLLCI	BootULCI	Significance
Environmental concern	Attitude toward recycling	Recycling behavior	0.106	0.055	0.160	Significant
Recycling knowledge	Attitude toward recycling	Recycling behavior	0.170	0.110	0.234	Significant
Moral norms	Attitude toward recycling	Recycling behavior	0.076	0.049	0.108	Significant

Note: indirect effects were estimated using bias-corrected bootstrapping with 5000 samples, mediation is considered statistically significant when the 95% confidence interval does not include zero.

## Data Availability

Data are available upon request due to restrictions (privacy and ethical reasons).
